# Supplementary material for: Tragedies, Fates, Furies and Fuels: Narratives of Individuals Bereaved by Suicide
Source: Int J Environ Res Public Health. 2022 Jul 18;19(14):8715. doi: 10.3390/ijerph19148715 (PMC9317914; doi:10.3390/ijerph19148715)
Supplement: Supplementary file 1 [file ijerph-19-08715-s001.zip › ijerph-1775548-supplementary.pdf]

MAIN QUESTION	Primary Prompt/s	Secondary Prompt/s
**1. I would love to hear about the meaning behind the photos you have taken/provided. Can you please tell me about them?**	**How do these photos relate specifically to your loved ones' experience?**	
	**What do these photos mean to you?**	In what way is this photo important to you?
		Tell me a little bit about this photo and why you took/chose it
	**Can you talk a little bit about what these photos represent for you?**	In what way is this photo important to you?
		Tell me a little bit about this photo and why you took/chose it
	**What does this photo say about your loved ones' experience?**	What story does the photo tell? What is the story behind this photo?
		How do you feel when you look at your image?
		What experiences does this photo remind you of?
	**Why have you chosen these photos to talk about?**	How is this photo significant to you?
		Why did you choose not to include the other photos?
	**When was this photo taken/where was it taken/?**	

For the photo elicitation component of the interview; participants in the **bereaved group** will be given the choice to take photos pertaining to their own experience/s of how their loved one was let down by the system and/or the way they perceive their loved ones' experience/s of being under the radar.

MAIN QUESTION	Primary Prompt/s	Secondary Prompt/s
**2. Can you please tell me about the experience/s of your loved one?**	**Can you share what you know of your loved ones' experience of living with thoughts of wanting to end his life?**	Was he forthcoming with these thoughts?
	**How did he experience thoughts of wanting to end his life?**	
		What was the frequency of these thoughts?
	**Could you tell me about the time or times in his life when he experienced thoughts and feelings of wanting to end his life?**	What happened in the lead up to these experiences?
		What happened during?
		Did he speak to you about these?
		What was going on in his life at the time?
	**Please describe how he was feeling during his experience(s)**	Were there things that you noticed about your loved one?
		How long did he have these thoughts for?
		Were there things that other people in his life noticed?

MAIN QUESTION	Primary Prompt/s	Secondary Prompt/s
**3. What kind of support would have been helpful for your loved one?**	**What other kinds of support can you think of that might have been helpful?**	Emotional support?
		Tangible support (aids and services)?
		In what way would the support have been helpful?
	**Why wasn't he able to get this support?**	What made it difficult for him to get this support?
		What concerns did he have about seeking support? If any?
		Would you say the barriers were internal or external?
	**Why do you think this kind of support would have been helpful?**	
	**Would formal mental health services (private/public - psychiatrist, psychologist, social worker, a mental health service) have been helpful? Probe for knowledge base in this area...**	How do you think he would have responded if he saw a mental health professional?
		Did he ever talk about accessing formal mental health care? Barriers? Desire?

MAIN QUESTION	Primary Prompt/s	Secondary Prompt/s
**4. If you could envisage something that would really have worked for your loved one in difficult times, what would that look like?**	**What do you think would support others during similarly difficult times?**	
	**What would seeking help have looked like for him?**	Thinking about your journey and experiences, at what point would you have liked to receive this support? When do you think this help would have been most useful?
	**Who, if anyone, would have been involved in giving him that support?**	
	**How – in your opinion - would he liked to have let people know that he needed support?**	
	**What do you think is missing in existing services/supports?**	What would you have liked to have been available to your loved one during this time?
		Thinking about when he had to manage hard times on his own, or with less support than he would have liked or needed, what would have been useful?
	**Is there anything else you can think of that would have helped him to navigate these difficult times, if there were no real-world constraints?**	Where would he have gone first to seek support? How would he have done that?

MAIN QUESTION	Primary Prompt/s	Secondary Prompt/s
**<u>Concluding Question:</u>**<br><br>**In what ways do you feel you have been able to share your loved ones' experience in this interview?**	**Is there anything else you would like to share that we may have missed?**	Thinking about everything that we have discussed, is there anything else you would like to revisit?
	***Summarise the study goals [1] *<br>Is there any other knowledge you think would be useful for us to have?**	What further information, if any, would you like to share that will help us meet the goals of this project?
		What do you feel this interview was not able to capture?
		How do you feel we can improve this interview for others?

[1] *"The purpose of this study was to learn more about the individuals' experiences of men who have ended their lives, and what kinds of support may have been helpful or not helpful for them. This information will be used to help us design a different kind of support that might help people to navigate these feelings. Keeping in mind the purpose of the study, is there any other knowledge you think would be useful for us to have"?*
